# Insights from older adults in North Carolina: Understanding social determinants of health through focus groups

**DOI:** 10.1080/29944694.2026.2645838

**Published:** 2026-03-27

**Authors:** Zachary Schrank, Nehemiah Stewart, Ritushree Dutta, Lauren Bedard, Claire Evans, Pavitra Madala, Aiden Pearson, Amy Zhang, Simran Sehgal, Bisma Qureshi, Kelly Carey-Ewend, Tooba Rashid, Lindsay Wilson

**Affiliations:** a UNC School of Medicine, Chapel Hill, North Carolina, USA;; b Department of Medicine, UNC SOM, Chapel Hill, North Carolina, USA

**Keywords:** Older adults, social determinants of health, focus group, qualitative research, older adults, North Carolina, rural, urban

## Abstract

Older adults experience unique social determinants of health (SDoH) that profoundly impact their health outcomes, but these factors are often overlooked in their healthcare. SDoH are the socioeconomic conditions in which people live, which drive over 60% of health outcomes. This qualitative study aimed to understand older adults’ perspectives on the most significant SDoH in their lives, as well as what their healthcare providers may be overlooking. We conducted focus groups with individuals aged 55 and older across three North Carolina counties (Caswell, Chatham, and Orange Counties) to assess how different community contexts influence SDoH. Financial stability and paying for healthcare were discussed similarly in all counties, as were provider overburdening, healthcare center inefficiency, and insurance company influence in limiting healthcare access. Caswell and Chatham participants discussed education access to navigate healthcare systems, whereas Orange and Caswell counties discussed transportation and housing concerns. Additionally, Orange and Chatham participants described senior socialization for health. All groups felt that time and good relationships with providers were essential for positive outcomes and facilitating SDoH discussion. These insights from the voices of older adults shed light on both common and diverse needs of these individuals across various communities and can guide healthcare and policy initiatives to improve health outcomes in this population.

## Introduction

In recent years, there has been a growing recognition of the profound impact that social determinants of health (SDoH) exert on individuals’ overall well-being and health outcomes (Braveman & Gottlieb, 2014; Hood et al., 2016; McGinnis et al., 2002; Morelli, 2023). The World Health Organization defines social determinants of health as the conditions in which people are born, grow, live, work, and age, encompassing factors such as socioeconomic status, education, physical environment, employment, and social support networks (Frank et al., 2020; Healthy People, 2030). National efforts to improve well-being of all citizens have incorporated SDoH into nation-wide health initiatives. For example, Healthy People (2030) is a national strategy to improve health of all Americans by prioritizing public health efforts, and this initiative bins SDoH into the five domains of Economic Stability, Healthcare Access and Quality, Education Access and Quality, Neighborhood and Built Environment, and Social and Community Context (Healthy People, 2030). These factors have been shown to significantly influence health outcomes, often more so than access to medical care alone (Hood et al., 2016; Mannoh et al., 2021; Marmot, 2005; McGinnis et al., 2002; Walker et al., 2014). Among the various demographic groups, older adults (individuals aged 55 and above) represent a population particularly susceptible to the effects of social determinants of health due to their unique life circumstances and vulnerabilities (Adler & Stewart, 2010; Fulmer et al., 2021; Hash & Hicks Patrick, 2024; Social Determinants of Health and Older Adults, 2024; Yamada et al., 2015). While the term “older adults” typically references individuals aged 65 or older, we include individuals between the ages of 55 and 64, as well. Adults between the ages of 55 and 64 are often in a transitional phase where early-aging health concerns begin to appear. We believe that including individuals 55 to 64 years of age captures perspectives on healthcare access and challenges that may differ from those of younger adults but are prospectively aligned with those 65 and older (Amarya et al., 2018; Preston & Biddell, 2021).

While research has highlighted the importance of addressing SDoH in healthcare delivery, there remains a gap in understanding how these factors are perceived and experienced by older adults themselves, as well as how healthcare providers are addressing them in clinical practice (De Marchis et al., 2022). Older adults may encounter distinct social determinants that impact their health outcomes differently, especially when considering the geographic regions in which they may reside. Factors such as access to transportation, community resources, housing stability, social isolation, and food insecurity are among the myriad of challenges that older adults face in their daily lives, which can significantly influence their health status and healthcare utilization (Weeks et al., 2023). The intersectional impact of age and socioeconomic status on health outcomes is thus often particularly severe, and this intersectionality can be amplified by the significant disparities in resources and access that exist in rural versus urban areas (Jaffe, 2015; L et al., 2002; Mattos et al., 2019; Smalls et al., 2023).

This qualitative study aimed to explore the lived experiences of adults older than 55 years living in three counties of varying ruralness in North Carolina (Caswell, Chatham, and Orange Counties) regarding the SDoH that they perceive as most impactful on their health outcomes. Furthermore, we aimed to examine the extent to which older adults feel their healthcare providers are addressing these social determinants in their clinical encounters. By conducting focus group discussions with older adults from different geographic locations, we sought to gain insights into the specific social determinants affecting their health and well-being in the context of the communities they reside in.

Understanding the nuances of social determinants of health among older adults is critical for informing targeted interventions and policy initiatives aimed at improving health equity and outcomes in this population (ASPE, 2024; Palmer et al., 2019). By elucidating the similarities and differences in the experiences of older adults across different counties in North Carolina, this study contributes to a broader understanding of how social determinants shape health outcomes in various communities. Through the voices of older adults themselves, we aim to shed light on overlooked factors that warrant greater attention in healthcare delivery and policy planning to better support the health and well-being of older adults.

## Methods

### Study design

The multidisciplinary research team consisted of a combination of student researchers at various levels of training led by a board-certified geriatrician at an academic medical center. Research coordinators and focus group moderators were upper-level medical students interested in geriatric medicine. Undergraduate, medical, and graduate students studying social work or public health served as focus group scribes and participant recruiters. We performed five qualitative focus groups consisting of older adults above the age of 55 in three senior centers across three counties in North Carolina during 2023: Caswell, Chatham, and Orange Counties. Table 1 summarizes the key socioeconomic differences between the three counties sampled in this study. At least one focus group was conducted per county with no participants being involved in more than one focus group. Participants’ informed consent was obtained verbally, after which demographic data were collected individually through a verbally administered survey (Table 2). De-identified qualitative data were then generated through a semi-structured focus group. We applied inductive thematic analysis to these qualitative data to ensure findings were not influenced by the preconceptions of team members and rather arose objectively from the data (Akhavan et al., 2022; Braun & Clarke, 2006; Kiger & Varpio, 2020; Malik et al., 2025). This study was approved by the University of North Carolina (UNC) institutional review board for all participating sites under IRB # 23–0048. A waiver of written consent was obtained for this study to eliminate use of any identifiable information from participants.

### Demographics of included counties

#### Study setting and participants

**Figure 1.**
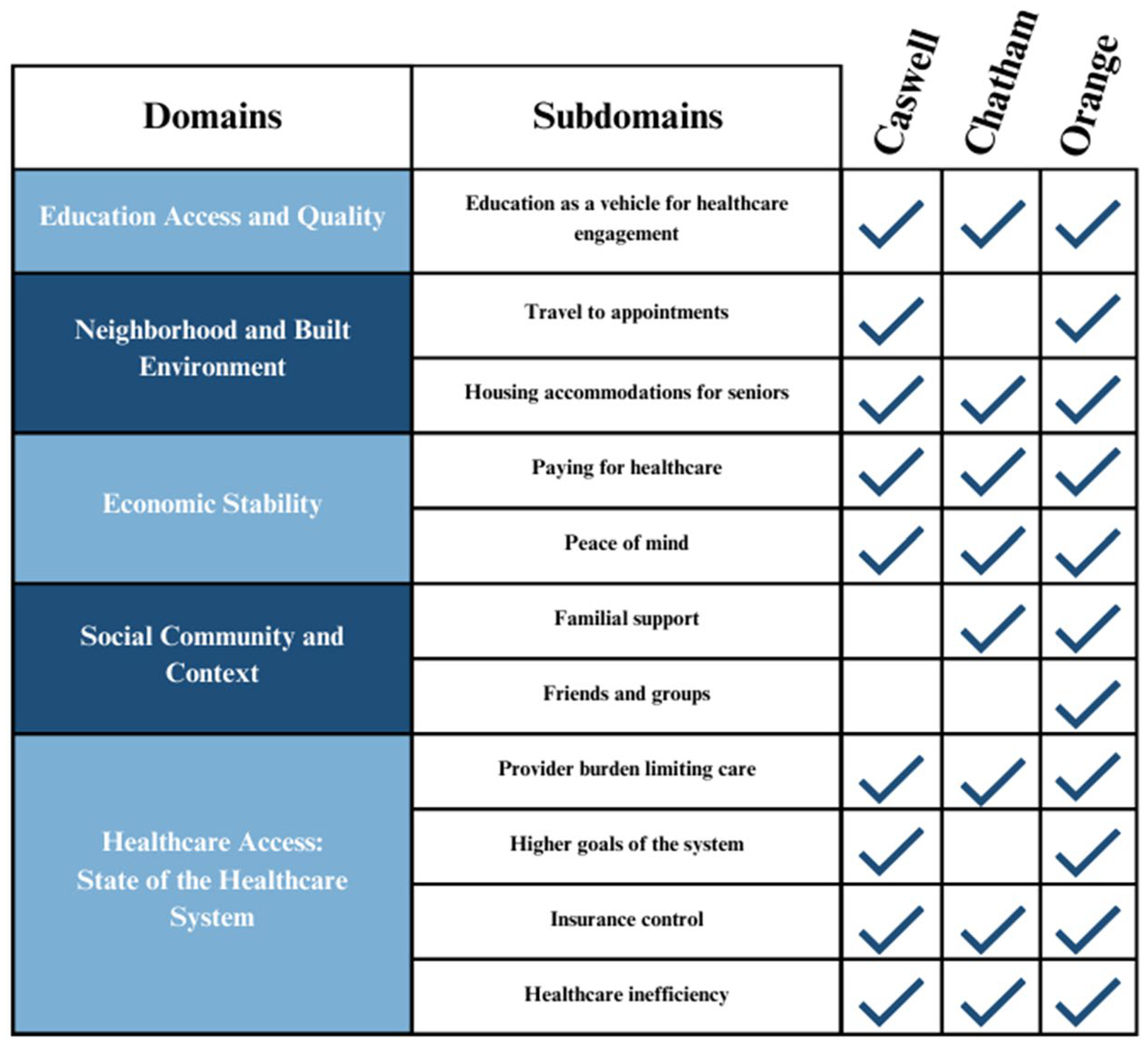
Summary of SDoH domains discussed in focus groups in each county.

Older adults were recruited to participate in the focus groups from senior centers in Caswell, Chatham, and Orange Counties to capture geographic and rural vs. urban diversity. Participants were recruited via in-person recruitment events held at the senior center at least 1 week prior to the date of the focus group, as well as via flyers emailed to community-based organizations in the area. The focus groups occurred in March, April, May, and November of 2023, with two held in West Chatham Senior Center (Chatham County), two in Seymour Senior Center (Orange County), and one in Caswell Senior Center (Caswell County). Focus groups in Caswell County were limited to one session due to limited turnout of prospective participants. The focus groups were conducted in-person at each location, with the demographic survey administered individually in private rooms before all participants were brought together for the focus group. The demographic survey lasted approximately 5 to 10 minutes for each participant, and the focus groups lasted approximately 60 to 80 minutes. Due to the flow of discussions within the focus groups in addition to restrictions placed by time, some topics were not discussed at certain focus groups, which is reflected in Figure 1. Participant were compensated for their participation in the study with a small gift bag containing stationary and confectionaries.

#### Data collection and processing

Focus groups were chosen in order to enrich the discussion through feedback and interactions between participants. For each focus group, a member of our team served as moderator to lead the participants through each question, facilitate discussion, and promote group process. Two to three additional team members were also present at each focus group to assist in administering the demographic survey and to directly document the conversation of participants. The WHO definition of SDoH and associated domains were provided at the administration of the demographic survey and referenced during the focus group (Healthy People, 2030). Participants of each focus group were asked three questions assessing perceptions of salient SDoH in their lives, what SDoH their doctors address well, and what SDoH their doctors do not adequately address. We informed participants that all information discussed during the focus group was confidential and that all data would be deidentified to encourage discussion and foster a feeling of safety amongst the group. We also clarified that no participant was obligated to answer any of the questions asked.

#### Analytic methods

Qualitative data were analyzed using NVivo software. Inductive thematic analysis was employed to ensure conclusions were data-driven and free of bias (Braun & Clarke, 2006; Kiger & Varpio, 2020). Braun and Clarke (2006) define thematic analysis as “a method for identifying, analyzing, and reporting patterns (themes) within [qualitative] data.” (Braun & Clarke, 2006) The inductive and deductive nature of thematic analysis is analogous to bottom-up and top-down analysis of qualitative data, respectively. Inductive analysis to synthesize conclusions in the focus group data helped identify intra- and inter-group themes and similarities (Proudfoot, 2023). Focus group data were independently documented and analyzed by a minimum of two different team members to verify the authenticity of the data. All transcripts were cross-checked within each focus group. Central themes of social determinants of health were compared amongst and between counties using a master list of codes and themes. Regular team meetings were held to discuss and check team members’ assumptions.

## Results

### Demographics of focus groups

Summarized demographics of focus groups provided in Table 2.

#### Most significant SDoH

SDoH were presented to participants and discussed in the context of the Healthy People 2030 framework (Healthy People, 2030). Participant discussion in focus groups was analyzed using this framework, which guided identification of subdomains under the main domains of Economic Stability, Healthcare Access and Quality, Education Access and Quality, Neighborhood and Built Environment, and Social and Community Context. Domains and subdomains discussed by participants in each county’s focus groups are summarized in Figure 1.

#### Domain: Economic stability.

Paying for healthcare. Participants from all counties discussed the impact of financial status on both access to and quality of healthcare. Participants from Chatham and Caswell Counties stated that their limited finances dictate their ability to receive care, as well as the type of care they receive. Participants from both counties also described the inability to afford insurance with rising insurance costs, which likewise significantly impacts their care. One participant specifically mentioned a friend foregoing certain procedures due to a lack of insurance.

“She’s giving up her doctor’s appointments because she doesn’t have the extra $20 to pay that part of the bill.”

Many participants also addressed the increasing cost of healthcare and how this creates concern around whether they will be able to obtain the procedures and prescriptions they need, despite one participant describing their medication as “the only thing that keeps (them) going.” Participants in Caswell County said they “gave up” their doctor’s appointments or had to “cut out something else” due to an inability to pay, indicating that they had to there was a tradeoff at play in order to avail healthcare and medications. It was evident that on the list of financial priorities, healthcare fell to the bottom, as participants from Caswell County navigated their costs of living given the significantly lower median income in the county (Table 1).

“If you really want it, you pay for it. And you cut out something else.”

One participant in Orange County mentioned difficulties affording necessary lab tests, and a second participant described having to “look for every possible coupon” in order to afford their medication. A third participant described their experience with the increasing cost of healthcare regarding heart surgery.

“In 2014, I had heart surgery - it wasn’t a surprising bill. I just had it again in March, but the copay this time was much higher. The prices for the surgeon and anesthesiologist have really risen.”

Participants from all counties also referred to financial stability as providing peace of mind and contributing to their overall well-being. One participant from Chatham County mentioned the negative impact on mental health that a lack of financial security can cause.

“Financials can wear you down if you don’t know how you will pay.”

A participant from Orange County remarked that one facet of having financial stability is having the peace of mind to cover health expenditures.

“Part of having money is having the peace of mind that, if my medicine is too expensive, I’ll still be able to use it.”

One Caswell County participant described their gratitude and comfort in being able to afford their husband’s chemotherapy treatments, which were reduced in cost due to his employment at a healthcare center. Another participant in the Caswell group stated that their healthcare is impacted not only by their individual financial status but also by the cost of other necessities overall, such as fuel prices and travel expenses. They expanded on this point by saying that the uncertainty of what will happen to the economy in the future causes them significant stress.

##### Domain: Healthcare access and quality.

Healthcare access was discussed in the context of several themes. Two major themes emerged: concerns with the state of the healthcare system and a lack of resources. From these major themes, several sub themes emerged.

###### State of the healthcare system.

Barriers to care caused by the state of the healthcare system were a significant theme brought up across all focus groups. However, the participants addressed the state and limitations of the healthcare system through different contexts, with participants from each county emphasizing specific factors that impede their ability to receive quality patient care.

###### Burden on providers.

Participants in all counties attributed delays and reduced healthcare access to pressures and limitations imposed on providers by healthcare institutions. In Orange and Chatham Counties, participants detailed how providers are under an imposed timeline by the healthcare system that enforces short clinical visits, limiting time with patients. A participant in Orange County noted that this timeline pressure takes the provider’s focus off the patient.

(Referencing short clinic visits) “That is all you are going to get. (Providers) ain’t got time for that.”

Furthermore, a participant in Orange County expressed that healthcare centers are understaffed, and providers are overworked, resulting in less productive patient interactions. A participant in Caswell County endorsed the impact of the COVID-19 pandemic on healthcare staff in the county, which all group members agreed upon. They discussed how COVID-19 reduced the staffing of healthcare centers, placing an increased burden on remaining providers that limited the ability to deliver care.

###### Higher goals of the system.

The participants in Orange County noted that they perceived the healthcare system and the care it delivers to be significantly influenced by factors other than patient needs. One participant put it quite plainly:

“It’s all about politics.”

This participant went on to express their belief that healthcare systems place greater emphasis on productivity and patient volume than patient care. Participants also felt that providers may be prioritizing financial gain over patient well-being. They believed these maligned priorities encourage workers to spend more time doing unproductive tasks without consequences because the system protects healthcare workers over patients. Similarly, Caswell County participants expressed that their rural status causes their care to drop even lower in priority, making it more difficult for them to navigate the healthcare system. In addition, older adults in Caswell County shared concern over the perspective that providers are more likely to provide better care to younger patients than older patients. Participants believed that treatment for age-related health conditions merited less attention and care due to the perceived lack of potential from treatments for older patients. One participant explicitly mentioned:

“Why does it mean because he’s older and has medical conditions that his life isn’t worth as much?”

Another participant shared a grim summary of the rhetoric they had been hearing regarding the priorities of the healthcare system.

“I hear a lot. ‘They do not care about us. The government does not care about us. Nobody cares about us. We’re just going to die.’”

###### Insurance control.

Participants across all counties expressed a negative view of insurance control over access to quality healthcare. Participants in Chatham County highlighted that a patient’s insurance and financial status inherently influence whether they receive care and what type of care they receive, preferring to actively manage their health rather than solely relying on providers. One participant succinctly summarized:

“What insurance you have dictates your workup.”

Conversely, participants in Orange and Caswell highlighted the negative impact of insurance companies’ practices on affordability and accessibility. Specifically, those in Orange County suggested that insurance companies are exploiting patients for profit and contributing to the increasing inaccessibility of healthcare due to rising costs. In Caswell County, participants described a belief that insurance companies have an overwhelming influence over healthcare access, especially given the steep copays of medical visits and procedure costs.

###### Healthcare inefficiency.

Across all counties, participants reported inefficiencies in the local healthcare system that impeded access to quality care. Participants from Chatham and Orange Counties criticized the lack of consistent care; continuous referrals to other specialists and even booking appointments exacerbated the issue of time delays in healthcare, further contributing to healthcare inefficiency. Chatham County participants particularly emphasized the issue of time delays in healthcare, with some participants discussing that they could not use their Urgent Care Centers due to significant wait times. Some Orange County participants believed that inefficiency in healthcare is due to providers asking repetitive questions about information they already have access to, leading to wasted time or a lack of thoroughness in care that contributes to more time delays. Furthermore, participants in Orange and Caswell Counties emphasized the effect of COVID-19 on the healthcare system, which led to its own healthcare inefficiencies. Specifically, the pandemic generated major understaffing issues that clinics have not recovered from, relying on a smaller care team to deliver timely and comprehensive care.

###### Lack of resources.

In Caswell County, participants described situations in which they struggled to access sources of vital information specific to their needs, particularly regarding prescription prices. Participants in Chatham County also emphasized the need for more clinics in rural areas and discussed how few available clinics increases health burdens on the available providers in the area. A Caswell participant expressed that resource scarcity was a major driver of poor health outcomes rather than quality.

“I don’t think our healthcare here is awful by any means, I just think it’s the struggle of needing more.”

##### Domain: Education access and quality.

Education as a vehicle for healthcare engagement. Participants from Chatham County and Caswell County spoke about education access and quality as a means to access and understand their healthcare. A participant from Chatham County stated that one needed to be educated enough to know “the system” when speaking about paying medical bills and how to express personal needs to providers. Another participant expanded on this, stating that some do not have the education to know how to get healthcare, citing a lack of knowledge of free clinics as an example. One participant went so far as to say that education access and quality were the most important factors of the five SDoH domains discussed, and that the other domains “fall into” education. Participants in the Caswell County focus group shared the sentiment that a lack of knowledge about available local healthcare resources is a barrier, though they did not specifically attribute this to a lack of education. Interestingly, a participant in the Caswell County focus group described benefits and drawbacks of education in navigating healthcare. They endorsed that education can indeed be helpful when navigating the healthcare system but also stated that this education can be detrimental when you possess more medical knowledge than what providers actually engage you in regarding your care.

“And sometimes education is really great, but if you know what is going on in the medical field, it’s kind of a hindrance to know more than what doctors think you know.”

##### Domain: Neighborhood and Built Environment.

Travel to appointments. Participants from Orange and Caswell Counties discussed how limited transportation options hindered the ability to attend healthcare appointments. Participants in Orange County mentioned this obstacle only briefly when initially asked about major SDoH impacting their health, with one participant also citing challenges traveling between their home and the senior center since they stopped driving. Participants in Caswell County, however, described challenges with transportation faced by their community at large. Caswell participants cited a lack of public transit options in the community limiting the ability to get around and get to appointments. They also described the significant obstacles to using the limited available public transit. One participant detailed that, to use the transit system to attend an appointment, one would have to go during the day, on a day that the transit system happened to be going to the desired destination, and have the appointment completed by noon. Furthermore, participants stated that people in the community will offer rides to older adults to attend these appointments but will take advantage of senior adults by charging them $20-$40 to attend the visits they need. Despite this, participants stated that most people in the community need to travel significant distances for appointments with healthcare providers due to a lack of dental clinics or “regular doctors” (primary care providers) nearby.

###### Housing accommodations for older adults.

Participants in Orange and Caswell Counties discussed themes of housing accommodations and housing conditions as critical for the health of some older adults. An Orange County participant shared that they live in a nursing home with handrails on the walls for walking assistance. They stated that they have friends in their 50s and 60s aging in their homes without any plans for additional home modifications or safety measures, and this participant felt that these friends were not adequately prepared for aging in place. Other participants of the Orange County group endorsed that their home provides them with a sense of security and comfort. In contrast, a participant from Caswell County described examples of older adults living in poor quality housing and conditions in their community.

“These older adults are living in homes that are falling down around them with no help.”

This participant went on to detail their frustration with this shared situation amongst several senior community members and that their rural county does not receive the support from the state government that is truly needed to support them, which significantly impacts the health of these older adults in the community.

##### Domain: Social and Community context.

Familial support. Participants from Chatham and Orange Counties discussed the role of familial support as a determinant of health. Chatham County participants described family members as being able to identify health issues missed by providers and attributed this to family members being “vigilant” and knowing the “mannerisms” of their loved ones.

“I think your family watches you; they watch how I get up, all that stuff. If they see something different, they usually say something.”

Orange County participants emphasized the significance of familial support regarding senior socialization, particularly during the COVID-19 pandemic. One participant stated that they were never alone during the pandemic since their daughter and grandchildren lived in the same residence. Another participant noted that they would go into a “deep depression” if they did not have their wife during this time. Other participants in Orange County reported feeling a lack of familial support during the pandemic despite being able to connect via online platforms, saying “it wasn’t the same.” Several participants associated this lack of social connection during the pandemic with a decline in mental health, with one citing this as the cause of their significant weight gain.

###### Friends and groups.

Participants in Orange County also discussed the importance of friends and social group memberships in the context of senior socialization. They stated that face-to-face socialization is extremely important and that it is crucial to socialize not only with other older adults but with a large age range. Participants cited church and small groups as points of connection with others. Several mentioned a lack of socialization causing “a problem” or depression, largely exacerbated by the COVID-19 pandemic. Another participant described the limited socialization in their current living situation.

“I live alone, very rural area, and the socialization is just nonexistent. I come to the senior center, I take classes, and nothing really seems to fit the bill.”

###### SDoH addressed and overlooked by providers.

In general, participants largely focused on factors leading to positive or negative experiences with their providers and how these factors facilitate discussion of SDoH, rather than what SDoH were specifically addressed or unaddressed by providers. The factors described largely fell under those leading to favorable versus unfavorable experiences. Sufficient time and personal relationships with providers facilitated positive experiences, whereas a sense of dismissal by the provider on account of their age led these older adults to have negative experiences.

##### Factors leading to favorable experiences.

Time with provider. Participants in all three counties discussed how time is a significant factor in creating favorable experiences with their providers. Focus groups in all counties associated good providers with those who spend more time with their patients and do not seem to be in a rush. Participants discussed how providers who take more time with their patients foster an environment where personal relationships can be built and health needs and SDoH can be addressed properly. There was also a consensus from all three counties that they would like to have more time with their providers to discuss health needs and that more time with providers leads to better healthcare experiences. Older adults in Chatham County discussed how some healthcare systems limit the time that providers can spend at each appointment and how sometimes that duration is not enough to adequately address health concerns, especially SDoH factors. Participants in Orange County and Chatham County understood that providers’ time can be limited by systemic factors but discussed how providers should make better use of the time available. One Orange County participant noted:

“I understand that doctors are seeing so many patients and they do not have much time. They should see the chart before you see the patient. They come in and see the chart in the patient room and ask repetitive questions about things I have already sent them before.”

A Caswell participant shared a similar sentiment regarding providers’ use of time:

“I think a really good doctor is when they come into the room it’s like they have all the time in the world. They’re not trying to get through the visit really quickly.”

###### Personal relationships with provider.

Participants across all counties emphasized how having a personal relationship with their provider and a feeling of connectedness were important factors in having positive experiences with providers. A Caswell County participant equated a physician who feels like a “partner” in healthcare to a physician who is effective at their job. All countries labeled good providers as those who are caring, compassionate, and take the time to form personal relationships with their patients. When participants had personal relationships with their providers, they felt that their health needs and social factors impacting their health were better addressed. In Caswell County, participants discussed how providers in rural areas that form these relationships are more in tune with the challenges faced by the community at large.

“(They) have doctors that go above and beyond to help individuals because they do know the struggles here.”

##### Factors leading to unfavorable experiences.

Patient dismissal. Older adults in Orange and Caswell Counties discussed a sense of dismissal from their providers due to their status as older adults. Caswell participants talked about how they feel that older patients are valued less in healthcare because providers believe older adults are less capable of understanding their health. Participants then went on to express concern that resources are often preferentially allocated to younger patients. Older adults at Caswell County believe this has a significant negative impact on their quality of care. Participants in Orange County described situations in which they felt that their input was ignored by providers and brushed off, and thus not truly getting to the root of the problem.

## Discussion

This qualitative study sheds light on the various SDoH impacting older adults in North Carolina, some of which are shared across rural and more urban counties. Under the domain of economic stability, participants in all counties discussed obstacles in both paying for healthcare, as well as the peace of mind that financial stability provides. In particular, older adults from all counties described instances of having to forego important medical exams, procedures, or medications due to an inability to pay. Several participants detailed how vital these medications and procedures are to their health, explaining that they had to make sacrifices or cut corners in other aspects of their lives to afford the care that they needed. Indeed, other studies and surveys have shown a similar trend in older adults and cancer patients in the US (Association of Health Care Journalists, 2024; Gallup, 2024; Han et al., 2020; Wiltshire et al., 2022). This may thus be reflective of a rise in healthcare costs experienced by everyone in the US, though the limited income of older adults, who often rely on Medicare, social security, retirement funds, or familial support, may feel this rise in cost greater than other demographics. This may be further reflected in the “peace of mind” that financial stability provided to older adults from all three counties. Participants described a sense of relief in having the finances to pay for healthcare despite rising cost and conversely noted significant stress when facing difficulty covering healthcare cost, as well as other expenses such as fuel and cost of travel.

Concerns about affording healthcare frequently led to discussions about a perceived undue influence that insurance companies have over the healthcare system, impacting healthcare access for participants in all counties. Likewise, participants from all counties discussed several obstacles to healthcare access beyond financial complications. Though many SDoH were discussed with regard to their significant intersectionality, participants described challenges with the healthcare system at large. Participants from all counties noted a perceived “overburdening” of healthcare providers due to pressures imposed by healthcare administrators to maximize the volume of patients seen. The COVID-19 pandemic was discussed as a considerable exacerbator of this problem due to effects on understaffing, but older adults from all counties described this enforced briefness of appointments as limiting the care they receive. However, older adults from all counties also endorsed the importance of time with healthcare providers as a determinant of positive experiences with providers. Some older adults placed the onus on providers to prioritize more time with patients, whereas others seemed to blame this short visit time primarily on pressures of administration and the system. Furthermore, older adults from Orange County pointed out that some of their providers seemed motivated by financial gain as opposed to patient outcomes. Together, these factors may suggest a perception amongst older adults that the healthcare systems they engage with, as well as the groups influencing and controlling them, may not have their best interests in mind. Other studies have shown a similar sentiment in the US general population regarding financial priorities in the healthcare system, suggesting this may be a scoping perception in US healthcare likewise felt by older adults (Blendon et al., 2014; Jha et al., 2008). Additionally, this lack of prioritization may further compound for older adults, who often feel marginalized and unvalued in the medical field given their age (Kang & Kim, 2022; Wyman et al., 2018). Indeed, participants in Caswell County may have captured this sentiment when they described how healthcare resources are unduly allocated to younger patients and that older adults are often perceived as less capable of understanding their health and healthcare plans.

The descriptions of inefficient healthcare centers by participants in all counties may also speak to general challenges faced by similar centers in the US healthcare system, including delays in appointment bookings and referrals, understaffing, brief visits, and disorganized workflow. Studies have suggested potential contributing factors, including primary care workforce shortages, significant time spent on electronic health records, or administrative burdens (Herd & Moynihan, 2021; Nguyen et al., 2024). These obstacles have been exacerbated by the COVID-19 pandemic (Gertz et al., 2022; Sabetkish & Rahmani, 2021), which was noted by participants in this study. However, given that older adults face a higher prevalence of chronic and acute medical conditions, including requiring specialist care (Boersma et al., 2020; Esme et al., 2019; Lueckmann et al., 2021), the negative impact of these inefficiencies for older adults may be more severe. This problem may be further exacerbated for older adults living in areas with insufficient healthcare resources already. This concern is reflected by participants in the rural Chatham and Caswell Counties regarding a lack of healthcare information in their communities as well as insufficient clinics and other health resources.

Participants in some counties also discussed SDoH uniquely from other counties. Participants in the more rural Chatham and Caswell Counties described the significant impact that education access and quality have on health, with one participant stating that it is the most important SDoH. Interestingly, participants discussed education access and quality with regard to general knowledge about the healthcare system and resource availability in the community rather than formal education or medical knowledge. The average education attainment of Orange County is higher than that of Chatham and Caswell Counties (MyFutureNC, 2024), and participants in Orange County groups generally had higher educational attainment than the other two counties (Table 1). This may reflect that education access may not be at the forefront of SDoH considerations for individuals that do or have had greater access. This may further reflect a lack of healthcare presence in these more rural communities, limiting dissemination of educational materials and general knowledge about healthcare services.

Interestingly, limitations of transportation were discussed in both the more urban Orange County and rural Caswell County. However, while an Orange County participant mostly referred to increased transit difficulty because they stopped driving, the participants in Caswell County expounded how the comparatively limited transportation options in their rural community negatively impacted their health and left them vulnerable to exploitation. The discussion of housing quality in these two counties also demonstrated stark differences. Orange County participants largely described the importance of housing accommodations with age, and Caswell participants described general poor quality housing conditions faced by older adults in the rural community. These discussions highlighted notable differences in SDoH faced by older adults in these different community settings.

Additionally, the need for socialization and social supports was only mentioned in Orange and Chatham Counties. Older adults from both of these counties detailed the negative impacts of COVID-19 and the resulting isolation on their mental health, which was in part relieved by family as well as social groups such as those found at church. One participant noted that living alone in a rural area significantly exacerbated this problem; however, older adults in the rural Caswell County did not describe challenges with socialization. While the data provided by a focus group format is dictated largely by the flow of discussion, the absence of this factor in Caswell County may reflect a stronger support network in some rural communities (Buck-McFadyen, 2024; Debertin, 1996; Ziersch et al., 2009).

Participants across counties largely did not discuss SDoH overlooked or addressed by providers. When asked about SDoH overlooked and adequately addressed by providers, participants in all counties primarily discussed the importance of sufficient time, effective communication, and establishing a personal relationship with the provider as determinants of positive outcomes. Older adults felt these factors enhanced their ability to discuss their health and socioeconomic problems, while enhancing their sense of security in the care that they received. Many older adults, especially those in Orange County, endorsed that their providers address their health adequately; however, this may reflect that these older adults do not see SDoH as factors that their providers are supposed to inquire about and address. Indeed, a participant from Caswell County expressed that they did not see the relevance of SDoH to their health in general. This may suggest a lack of discussion about SDoH in healthcare encounters, but also a need for patient education regarding the importance of these factors in health. A survey of providers across the US by The Physicians Foundation showed that 80% of providers believe that addressing SDoH is necessary to improve health outcomes in the US (The Physicians Foundation, 2022), but data regarding patient perceptions of these factors is lacking. Future studies investigating the perceptions of different patient populations regarding the importance of SDoH may inform beneficial educational strategies that promote patient-provider engagement regarding SDoH.

This study has several limitations to consider. Reliance on scribe notes instead of recordings and transcripts may have limited the depth of data collected, potentially missing nuanced contextual factors and introducing interpretive biases. Varying group sizes influenced by participant availability may have introduced sampling biases, affecting the diversity of perspectives. Omitting race and ethnicity information in the demographic survey limits understanding of cultural influences on social determinants of health. Additionally, the self-selection bias of participants, likely skewed toward those more active in the community or with strong opinions, may limit the generalizability of findings.

Overall, this study offers valuable insights into older adults’ experiences with SDoH. It highlights several shared factors, including economic stability and obstacles imposed by the state of the healthcare system, that may be experienced by patients across demographics but exacerbated for older adults. The unique factors described by some counties may illustrate SDoH impacting older adults in light of their community context. These findings demonstrate a need for comprehensive SDoH assessments in community healthcare, as well as the potential utility of SDoH assessments informed by community or patient context, guiding future research and interventions aimed at addressing health disparities in older adults.

## Figures and Tables

**Table 1.** National, state, and county level demographics and socioeconomic statistics.

Factor	United States	North Carolina	Orange County	Chatham County	Caswell County
Population	331,449,281	10,835,491	150,626	81,624	22,807
Individuals Aged 55 and older	101,181,600	3,285,656	42,717	33,142	8,500
% Rural	20%	33.3%	29%	64%	99.8%
Unemployment Rate	3.6%	3.5%	2.9%	3.0%	3.6%
Limited Access to Healthy Foods	6%	8%	2%	5%	3%
Food Insecurity	14%	14%	12%	13%	15%
Severe Housing Problems	17%	14%	17%	13%	10%
Ratio of Patients to Primary Care Physicians	1330:1	1410:1	550:1	1070:1	4540:1
% Medically Uninsured Adults	11%	14%	9%	13%	15%

Data from 2017 to 2023 (Health Data, 2025; Census.gov, 2025).

**Table 2.** Participant demographics.

		Orange	Chatham	Caswell
		Frequency	Frequency	Frequency
Participants		**18**	**10**	**5**
Number of Focus Groups		**3**	**2**	**1**
Number Insured	Medicare	**12**	**4**	**2**
	Medicaid	**1**	**1**	**0**
	Humana	**7**	**1**	**1**
	Blue Cross Blue Shield	**2**	**1**	**1**
	United Health	**2**	**2**	**1**
	Aetna	**3**	**4**	**0**
	AARP	**0**	**1**	**0**
Number Uninsured		**0**	**1**	**0**
Education Level	No High School	**1**	**0**	**0**
	Some High School	**0**	**0**	**0**
	High School Diploma or GED	**0**	**4**	**2**
	Some College	**5**	**4**	**2**
	College Degree*	**3**	**2**	**0**
	Graduate Level and above	**9**	**0**	**1**
		Mean (Std)	Mean (Std)	Mean (Std)
Age		**75.4 (7.1)**	**74.4 (5.2)**	**71.6 (9)**
Confidence in understanding of own health (1–10)**		**8.7 (1.7)**	**8.9 (1)**	**8.4 (1)**
Confidence in understanding of finances (1–10)**		**8.6 (1.8)**	**8.9 (1.6)**	**9.8 (0.4)**
Confidence that healthcare provider understands your health needs (1–10)**		**8.3 (2.1)**	**8.9 (1.2)**	**8.2 (1.3)**
Perception of how much healthcare provider cares about your health (1–10)**		**8.2 (2.1)**	**9.3 (0.9)**	**8.2 (1.2)**
Knowledge of resources available for your health needs (1–10)**		**8.6 (1.4)**	**8 (1.5)**	**7.2 (1.6)**

Std, Standard Deviation,

*- Bachelor’s or Associate’s Degree,

**- (1–10) scale with 10 indicating highest confidence, perception, or level of understanding.

## Data Availability

The data that support the findings of this study are available from the corresponding author, ZS, upon reasonable request.
